# Fibrinogen deficiency in a dog - a case report

**DOI:** 10.1186/s12917-017-1110-8

**Published:** 2017-06-19

**Authors:** Franck Jolivet, Armelle Diquélou, Catherine Trumel, Simon Privat, Olivier Dossin

**Affiliations:** 1 Department of Clinical Sciences, ENVT, University of Toulouse, 31076 Toulouse, France; 2 IRSD, INSERM 1220, INSERM, INRA, ENVT, UPS, University of Toulouse, 31024 Toulouse, France

**Keywords:** Fibrinogen deficiency, Hypofibrinogenemia, Dysfibrinogenemia, Bleeding disorders, Dog

## Abstract

**Background:**

Among coagulation disorders, primary fibrinogen deficiency is very rare in dogs. It is divided into hypofibrinogenemia, afibrinogenemia and dysfibrinogenemia. Afibrinogenemia has been described in three dogs. There are, however, no published case reports of primary hypofibrinogenemia in dogs.

**Case presentation:**

A 1.5 year-old male German Pointer dog was evaluated for a locked-jaw syndrome associated with eye protrusion which appeared after a minor head trauma. Three months before the trauma, a persistent increase in coagulation times was detected by the referring veterinarian after a strong suspicion of snake envenomation. Apart for the primary complaint, physical examination was normal. A complete hemostatic profile revealed a moderately increased prothrombin time, activated partial thromboplastin times and a dramatically decreased fibrinogen concentration (0.34 g/L, reference interval [1.3–4.8 g/L]). Platelet count, plasma D-dimers and antithrombin, were all within the reference intervals and not consistent with a disseminated intravascular coagulation. Other possible causes of hypofibrinogenemia such as chronic hemorrhage and liver failure were excluded by laboratory work-up and imaging studies. Finally, antifibrinogen circulating anticoagulants were excluded using a dilution of citrated plasma from the pooled plasma of healthy dogs. These results supported a diagnosis of congenital fibrinogen deficiency and secondary retrobulbar hematoma and/or cellulitis. The dog’s condition improved rapidly after symptomatic treatment with corticosteroids and antibiotics. At the 1 year follow-up, the dog was clinically normal but a persistent hypofibrinogenemia (≤ 0.8 g/L) remained.

**Conclusions:**

Various clinical presentations may occur in canine primary hypofibrinogenemia which should be included in the list of coagulation disorders. Diagnosis should include fibrinogen determination by coagulometric and non-coagulometric methods to differentiate from dysfibrinogenemia. There is no specific treatment but care should be taken to prevent bleeding and trauma. Emergency management of bleeding episodes with cryoprecipitate is the treatment of choice.

## Background

Congenital disorders of coagulation are rare in veterinary medicine, and consist mainly of hemophilia A and B [1]. Other clotting factor deficiencies have however been described and can be a diagnostic challenge. Among these deficiencies, hypofibrinogenemia has seldom been described in dogs.

Fibrinogen, the soluble precursor of fibrin, is a key coagulation factor involved in both primary and secondary hemostasis, promoting platelet aggregation and clot formation. Fibrinogen is the most abundant clotting factor, with usual blood concentrations ranging from 1.3 g/L to 4.8 g/L in adult dogs (reference interval (RI) from the veterinary clinical pathology laboratory of the Veterinary Teaching Hospital of the University of Toulouse (VTH UT)). In human and veterinary medicine, three congenital fibrinogen abnormalities have been described: afibrinogenemia (absence of fibrinogen), hypofibrinogenemia (quantitative deficiency) and dysfibrinogenemia (qualitative deficiency) [2–6].

Humans with congenital hypofibrinogenemia are usually asymptomatic but may bleed when the hemostatic system is strongly solicited, for example in such situations as trauma or surgery [2].

This report describes a 1.5 year-old German Pointer dog with a protrusion of the right third eyelid and a locked-jaw syndrome associated with extreme pain on mouth-opening due to a congenital hypofibrinogenemia.

## Case presentation

A 1.5 year-old male German Pointer dog weighing 27 kg was referred to the VTH UT for a protrusion of the right third eyelid, and a locked-jaw syndrome associated with extreme pain when the dog tried to open his mouth. These signs had appeared 10 days earlier, after the dog was hit on the head by a ball. The dog had remained stable since then. History revealed a strong suspicion of snake envenomation 3 months earlier. At that time, the dog was presented in shock to a veterinarian with marks on the head suggestive of a snake bite. The laboratory workup revealed markedly increased prothrombin time (PT) and activated partial thromboplastin time (aPTT). The veterinarian started a symptomatic treatment [fluid-therapy (Ringer Lactate), corticosteroids (prednisolone, 1 mg/kg, IV, q 12 h), antibiotics (marbofloxacin, 4 mg/kg, IV, q 12 h) and K1 vitamin] and clinical signs improved within a few days. However, the PT and aPTT remained moderately increased after this suspected envenomation. One month later, a gingival wound appeared, requiring a surgical suture to stop the bleeding. On this occasion, PT and APPT were still markedly increased (PT >35 s, RI [12–19 s] and aPTT >200 s, RI [75–105 s]).

Upon admission at the VTH UT, physical examination showed an exophthalmia of the right eye associated with protrusion of third eyelid. The dog was reluctant to be touched on the head and opening the mouth elicited severe pain. Apart from this, clinical examination was unremarkable and a thorough ocular examination did not reveal any other abnormality. The ocular ultrasonography was consistent with a retrobulbar cellulitis with no image suggesting the presence of a foreign body. Because of the history of abnormal coagulation, the retrobulbar fine needle aspiration was postponed until the laboratory results were available.

An extensive laboratory profile, including complete hemostatic profile^1^, was performed at the veterinary clinical pathology laboratory of the VTH UT. Severe hypofibrinogenemia (0.34 g/L, RI [1.30–4.80 g/L] assessed by Clauss’coagulometric method), increased PT (15.0 s, RI [7.10–9.00 s]) and aPTT (19.3 s, RI [12.8–17.2 s]) were observed. All other results including buccal mucosal bleeding time, complete blood count (CBC), D-dimers (DDi), and antithrombin (AT) were within reference intervals (Table 1). The fibrinogen/fibrin degradation products (FDPs) value was however in the grey zone (between 5 and 20 μg/mL, RI [< 5 μg/mL]).

**Table 1:** Complete hemostasis profile

Variable	Value	Reference Interval
Buccal Mucosal Bleeding Time	3 min10 s	3–5 min
Platelet count	175,000 /μL	64,000–613,000 /μL
PT	15.0 s	7.1–9.0 s
aPTT	19.3 s	12.8–17.2 s
Fibrinogen	0.34 g/L	1.30–4.80 g/L
FDPs	5–20 μg/mL	<5 μg/mL
DDi	0.51 mg/L	<0.56 mg/L
AT	115%	109–195%

The hypofibrinogenemia was observed on repeated coagulometric measurements as well as by physical measurement using the Millar heat-precipitation method based on the ability of fibrinogen to precipitate at 56 °C [7]. This method confirmed the severe hypofibrinogenemia with an almost undetectable fibrinogen precipitation band.

A screening for possible causes of hypofibrinogenemia was performed. The normal liver profile, including a normal bile acid test, excluded liver failure (Table 2). The absence of clinical signs of disease conditions that are associated with disseminated intra-vascular coagulation (DIC) together with the absence of any other laboratory signs of hemostasis system activation (normal platelet count, DDi and AT) was not consistent with DIC. Chronic bleeding was ruled out by clinical, diagnostic imaging and CBC findings. To rule out the hypothesis of circulating anticoagulant interfering with the fibrinogen determination, a 1/1 dilution of the dog’s citrated plasma with pooled healthy dog plasma (PHDP) was performed. PHDP was prepared by pooling identically prepared plasma samples from 9 clinically healthy dogs. The fibrinogen concentration assessed by coagulometric method in the mixture was consistent with the expected calculated result (Table 3). This finding ruled out the presence of circulating anticoagulant with fibrinogen inhibitory properties. A congenital hypofibrinogenemia was hence diagnosed.

**Table 2:** Complete blood chemistry profile

Variable	Value	Reference Interval
Creatinine	75 μmol/L	44–133 μmol/L
Cholesterol	4.7 mmol/L	3.3–9.3 mmol/L
Triglycerides	0.4 mmol/L	0.2–1.3 mmol/L
Total Protein	76.1 g/L	48.0–66.0 g/L
Albumin	35.3 g/L	23.0–39.0 g/L
Alanine Aminotransferase	45 U/L	0.3–50 U/L
Alkaline Phosphatase	195 U/L	20–155 U/L
Gamma-glutamyltransferase	5 U/L	0.5–25 U/L
Total Bilirubin	1.8 μmol/L	1.7–12.0 μmol/L
Pre-Prandial Bile Acids	2.0 μmol/L	0–10.0 μmol/L
Post-Prandial Bile Acids	2.0 μmol/L	0–20.0 μmol/L

**Table 3:** Fibrinogen concentration after plasma dilution with PHDP

Sample	Fibrinogen concentration
PHDP	1.70 g/L
Patient	0.51 g/L
PHDP + Patient (1/1, V/V dilution)	1.13 g/L

Theoretical expected result for the dilution: 1.105 g/L

*PHDP* Pooled Healthy Dogs Plasma

Retrobulbar fine needle aspiration was not performed due to the high risk of puncture-induced hemorrhage. In the hypothesis of a retrobulbar cellulitis, the following treatment was administered: antibiotics (gentamicin 6.6 mg/kg, IV, q 24 h for 6 days, and amoxicillin/clavulanic acid 20 mg/kg, IV, q 8 h for 6 days, then 12.5 mg/kg, PO, q 12 h for 6 weeks) and corticosteroid therapy (prednisolone 0.3 mg/kg, IV, q 24 h for 6 days then 0.3 mg/kg, PO, q 24 h for 3 weeks, and finally 0.3 mg/kg, PO, every other day, for 2 weeks).

After 2 days of treatment, the protrusion of the third eyelid and the pain associated with mouth opening decreased and resolved after 2 weeks. One month later, the dog was completely normal. Two months later, the dog was stable but fibrinogen remained persistently below the reference interval (Table 4) and the FDP value was negative (< 5 μg/mL). Six months after the initial presentation, no clinical relapse was observed but plasma fibrinogen value remained low.

**Table 4:** Follow-up of fibrinogen concentrations

Test time	Fibrinogen concentration	RI
Admission	0.34 g/L	
1 month later	0.70 g/L	1.30–4.80 g/L
2 months later	0.51 g/L	

There is no specific treatment for congenital hypofibrinogenemia, other than plasma transfusion during acute bleeding crisis. It was therefore strongly recommended to avoid strenuous exercise or hunting with the dog to prevent trauma.

## Discussion

This case describes fibrinogen deficiency, an extremely rare congenital bleeding disorder in dogs [1, 8]. Very little data is published on the subject in veterinary medicine, with only three case reports on dogs with afibrinogenemia: a Bernese mountain dog [3], a Chihuahua [4], and a Bichon Frise [5]. In an international registry of animal hemostatic disorders, Dodds mentioned a case of dysfibrinogenemia in an inbred family of Borzois and a case of hypofibrinogenemia in a family of Saint Bernard dogs [6] but to our knowledge these cases have not been reported in detail in a peer-reviewed journal.

In human medicine, congenital fibrinogen deficiency represents 7% of rare congenital bleeding disorders, hemophilia-excluded [2, 9]. Three categories of fibrinogen deficiency have been described: afibrinogenemia when fibrinogen is not detectable; hypofibrinogenemia when the value is below the lower limit of the reference interval; and dysfibrinogenemia when the fibrinogen molecule is abnormal and dysfunctional. The only way to differentiate hypofibrinogenemia from dysfibrinogenemia in humans is a low fibrinogenemia assessed by coagulometry with a concurrent normal or subnormal fibrinogen concentration by an immunologic method. Unfortunately, such a method is not routinely available in veterinary medicine [10, 11]. Therefore dysfibrinogenemia was not completely excluded in our dog. However, because the fibrinogen concentration was similarly decreased with both coagulometric method and heat precipitation method, we concluded it was hypofibrinogenemia.

The clinical presentation of ocular signs in this case is unusual. Ocular ultrasonography was suggestive of a retrobulbar cellulitis with effusion. To confirm this diagnosis, cytological and bacteriological analysis of a fine needle aspiration of retrobulbar space is needed. In this case, the first hypothesis was a hematoma because the ocular problems started after the dog was hit on the head and the patient had a background history of sustained increase of coagulation times and abnormal bleeding following a benign gingival wound. However, the nature of the retrobulbar ultrasonographic abnormality could not be confirmed by fine needle aspiration due to the risk of bleeding. Given the extreme pain associated with jaw opening, a primary hematoma with secondary inflammation/infection was a possible hypothesis. The dog was therefore treated with antibiotic and anti-inflammatory drugs. The rapid clinical improvement after onset of the treatment in a dog, whose signs had been stable for the previous 10 days was consistent with this hypothesis.

When hypofibrinogenemia is detected, the first clinical step is to exclude all possibility of a preanalytic error and to confirm the fibrinogen value using another method such as Millar’s method. If the decreased concentration is confirmed, all other causes of decreased fibrinogen production or increased fibrinogen consumption need to be explored before considering a diagnosis of congenital hypofibrinogenemia. Liver function must be assessed because fibrinogen is produced by the liver. In our case, normal clinical examination and bile acid test excluded decreased hepatic fibrinogen synthesis. The second step is to rule out increased and sustained fibrinogen consumption by activation of the coagulation cascade, namely DIC or chronic bleeding, by laboratory work-up and diagnostic imaging. No systemic disease, bleeding or inflammatory site, other than the ocular bulging, was suspected in our dog. No signs of chronic blood loss were detected on CBC, and normal platelet count, AT and DDi concentrations were inconsistent with DIC.

In our patient, the only hemostatic abnormality other than hypofibrinogenemia was FDPs in the grey zone. FDPs reflect the action of the fibrinolytic enzyme, plasmin, on fibrin and fibrinogen whereas DDi appear only when fibrin is degraded by plasmin [12]. Decreased FDPs clearance due to a failing liver or kidney was excluded by the laboratory results. The FDPs value results could indicate primary hyperfibrinolysis (*ie* spontaneous activation of plasmin not triggered by coagulation activation). However, primary hyperfibrinolysis had not previously been described in dogs and was considered very unlikely in our case for other reasons including the absence of primary diseases causing hyperfibrinolysis in humans, such as liver cirrhosis [13] or bronchiectasis [14]. Moreover, if the severe hypofibrinogenemia in this dog had been caused by hyperfibrinogenolysis, FDPs would have been very high and not only slightly increased. Finally, the dog’s fibrinogen concentration remained low at the 1 and 2 month follow-ups which was not compatible with a primary hyperfibrinogenolysis that is not a persistent condition. Therefore, we hypothesized that the slight increase of the FDPs could be the consequence of the suspected retrobulbar hematoma.

Furthermore, the unusual context of this case’s clinical scenario made it difficult to determine whether the disorder was congenital or acquired. The onset of the condition, discovered by the veterinarian in association with a strong suspicion of snake bite, was initially confusing. It concerned an adult dog, with no previous coagulation profile evaluation or surgical history, making it impossible to determine if the coagulation disorders were present prior to this episode. According to the owner, the dog had been perfectly healthy which is consistent with the few cases of hypofibrinogenemia described in the veterinary literature [1, 8].

Because the PT and aPTT values were consistently above the reference intervals after the presumed envenomation, the hypothesis of circulating anticoagulants, such as antibodies that would have developed after envenomation and would be directed against fibrinogen, or would impair the coagulation reactions involved in the transformation of fibrinogen into fibrin, had to be investigated. The dilution method with a PHDP is a simple method used to explore circulating anticoagulants. It is easily performed in-house when coagulation times are sufficiently increased (at least 1.5 times the upper limit of RI), only requiring the measurement of PT or aPTT in PHDP, the citrated plasma of the affected animal and a 1/1 (vol/vol) mixture of the two. If PT or aPTT are not normalized in the mixture, it strongly suggests a circulating anticoagulant/antibody against a coagulation factor [15]. In our case, we chose to measure the fibrinogen concentration ([Fibrinogen]) using a coagulometric method rather than coagulation times. Because the fibrinogen concentration in the mixture was close to [PHDP Fibrinogen]/2 + [affected dog’s Fibrinogen]/2, we ruled out the presence of a circulating anticoagulant in the plasma of the affected dog. A possible limitation to this approach would be that serial dilutions might have been more sensitive to detect a circulating antibody if the optimal concentrations had not been obtained at the 1/1 dilution. However, in the only case report of circulating antibody against fibrinogen described in the dog [5], the fibrinogen concentration of the 1/1 mixture was more than 50% below the expected theoretical concentration. A circulating anticoagulant was therefore considered unlikely in our case. Once all the hypotheses had been excluded, the final diagnosis was consistent with a congenital fibrinogen deficiency.

Testing the parents and the siblings to assess coagulation abnormalities would have been interesting to investigate the disorder in the whole family and document a possible genetic/hereditary background. Unfortunately, this was not possible in our case.

Cryoprecipitate is the treatment of choice for fibrinogen deficiencies because it supplies high concentrations of fibrinogen in a low volume. Fresh frozen plasma is an alternative product when cryoprecipitate is unavailable. For patients with active hemorrhage and decreased PCV, a blood transfusion is also indicated. The target fibrinogen value is 1.0 g/L [16]. The half-life of fibrinogen is 4.2 days and relatively long compared to most coagulation factors [17]. Therefore, fewer transfusions are often sufficient to temporarily support haemostasis in such patients.

## Conclusions

This case is the first complete report of congenital hypofibrinogenemia in a dog. Moreover, it is an original presentation of bleeding disorders suggesting that clinicians should consider bleeding disorders in differential diagnosis of locked jaw syndrome or eye protrusion.

## Abbreviations

aPTT: Activated partial thromboplastin time
AT: Antithrombin
CBC: Complete blood count
DDi: D-dimers
DIC: Disseminated intra-vascular coagulation
FDPs: Fibrinogen/fibrin degradation products
PCV: Packed cell volume
PHDP: Pooled healthy dog plasma
PT: Prothrombin time
RI: Reference interval
VTH UT: Veterinary Teaching Hospital of the University of Toulouse

## Footnotes

1 Sta-Compact, Diagnostica Stago, France.
